# Has Shouldice Repair in a Selected Group of Patients with Inguinal Hernia Comparable Results to Lichtenstein, TEP and TAPP Techniques?

**DOI:** 10.1007/s00268-017-4433-5

**Published:** 2018-01-03

**Authors:** F. Köckerling, A. Koch, D. Adolf, T. Keller, R. Lorenz, R. H. Fortelny, C. Schug-Pass

**Affiliations:** 1 Department of Surgery and Center for Minimally Invasive Surgery, Academic Teaching Hospital of Charité Medical School, Vivantes Hospital, Neue Bergstrasse 6, 13585 Berlin, Germany; 2 Hernia Center Cottbus, Gerhard-Hauptmann-Strasse 15, 03044 Cottbus, Germany; 3 StatConsult GmbH, Halberstädter Strasse 40 a, 39112 Magdeburg, Germany; 4 3Surgeons, Klosterstrasse 34/35, 13581 Berlin, Germany; 5 Department of General Surgery Wilhelminenspital, Medical Faculty, Sigmund Freud University, Montleartstrasse 37, 1160 Vienna, Austria

## Abstract

**Background:**

In the new international guidelines only the mesh-based Lichtenstein, TEP and TAPP techniques are recommended. This present analysis of data from the Herniamed Registry compares the outcome for Shouldice versus Lichtenstein, TEP and TAPP.

**Methods:**

Propensity score matching analyses were performed to obtain homogeneous comparison groups for Shouldice versus Lichtenstein (*n* = 2115/2608; 81.1%), Shouldice versus TEP (*n* = 2225/2608; 85.3%) and Shouldice versus TAPP (2400/2608; 92.0%).

**Results:**

The most important characteristics of the Shouldice patient collective were younger patients with a mean age of 40 years, a large proportion of women of 30%, a mean BMI value of 24 and a proportion of defect sizes up to 3 cm of over 85%. For this selected patient collective, propensity score matched-pair analysis did not identify any difference in the perioperative and one-year follow-up outcome compared with TAPP, fewer intraoperative (0.5 vs. 1.3%; *p* = 0.009) but somewhat more postoperative complications (2.3 vs. 1.5%; *p* = 0.050) compared with TEP and advantages with regard to pain at rest (4.6 vs. 6.1%; *p* = 0.039) and on exertion (10.0 vs. 13.4%; *p* < 0.001) compared with the Lichtenstein technique.

**Conclusion:**

For a selected group of patients the Shouldice technique can be used for primary unilateral inguinal hernia repair while achieving an outcome comparable to that of Lichtenstein, TEP and TAPP operations.

## Introduction

All the guidelines published to date recommend mesh-based surgical techniques for primary unilateral inguinal hernia repair because of the lower recurrence rate [1–6]. In the guidelines the open Lichtenstein, the total extraperitoneal patch plasty (TEP) and the transabdominal preperitoneal patch plasty (TAPP) techniques are recommended as best evidence-based options for repair of a primary unilateral inguinal hernia provided the surgeon is sufficiently experienced in the specific procedure [6]. The Shouldice hernia repair technique [7, 8] is the best non-mesh repair method [1, 2]. Other authors propose that mesh repairs should be abandoned and the Shouldice repair adopted since mesh repair is reported to be associated with an inguinodynia incidence of up to 21% [9].

To date, 380,000 operations have been performed at the Shouldice Hospital in Toronto [10]. On average each surgeon at the Shouldice Hospital operates on 700 patients per year. Using population-based, administrative health data a study of Ontario residents who had primary elective inguinal hernia repair at an Ontario hospital between 1993 and 2007 found that inguinal hernia repair at the Shouldice Hospital was associated with a significantly lower risk of subsequent surgery for recurrence than repair at a general hospital [11].

Likewise, the new international guidelines of the HerniaSurge Group [6] recommend a mesh-based repair technique for patients with symptomatic inguinal hernias. Whether a non-mesh technique is an alternative for mesh-based techniques in individual cases (e.g., young males with lateral hernia L1 and L2) is unknown and requires further study. Following these recommendations, the best non-mesh technique Shouldice should only be used in patients refusing a mesh repair and/or after a shared decision making and in settings with non-availability of meshes [6].

This present analysis now compares the perioperative and one-year follow-up outcome of cases of primary unilateral inguinal hernia repair documented in the Herniamed Registry [12, 13] which had been operated on with the Shouldice versus the Lichtenstein, TEP and TAPP techniques, respectively. As a robust approach for comparative effectiveness research in observational studies [14] we used propensity score matching to yield comparable groups for analyses. Previous findings from simulated data of observational studies showed that propensity score analysis could produce estimates that were less biased, more robust and more precise than with multivariable analysis [14]. A propensity score analysis aims to mimic randomization and thus deals with confounding bias [14]. Using the Herniamed Registry enables us to take many important potential confounding variables into account.

## Methods

The Herniamed quality assurance study is a multicenter, Internet-based hernia registry [12, 13] into which 524 participating hospitals and surgeons engaged in private practice (Herniamed Study Group) in Germany, Austria and Switzerland (status: March 7, 2016) have entered data prospectively on their patients who had undergone routine hernia surgery. In Germany, surgeons in private practice are not employed by a hospital. Rather, they operate on patients in outpatient/ambulatory surgical centers or hospitals for a fee. All patients signed an informed consent agreeing to participate. As part of the information provided to patients regarding participation in the Herniamed Registry and signing the informed consent declaration, all patients are informed that the treating hospital or medical practice would like to be informed about any problem occurring after the operation and that the patients have the opportunity to attend clinical examination. All postoperative complications occurring up to 30 days after surgery were recorded. At one-year follow-up, postoperative complications are once again reviewed when the general practitioner and patient complete a questionnaire. At the one-year follow-up, the general practitioner and patient are asked about any recurrences, pain at rest, pain on exertion and chronic pain requiring treatment. If a recurrence or chronic pain is reported by the general practitioner or patient, the patient can be requested to present themselves for clinical examination. One publication has provided impressive evidence of the role of patient-reported outcome for recurrence and chronic pain [15].

**Fig. 1:**
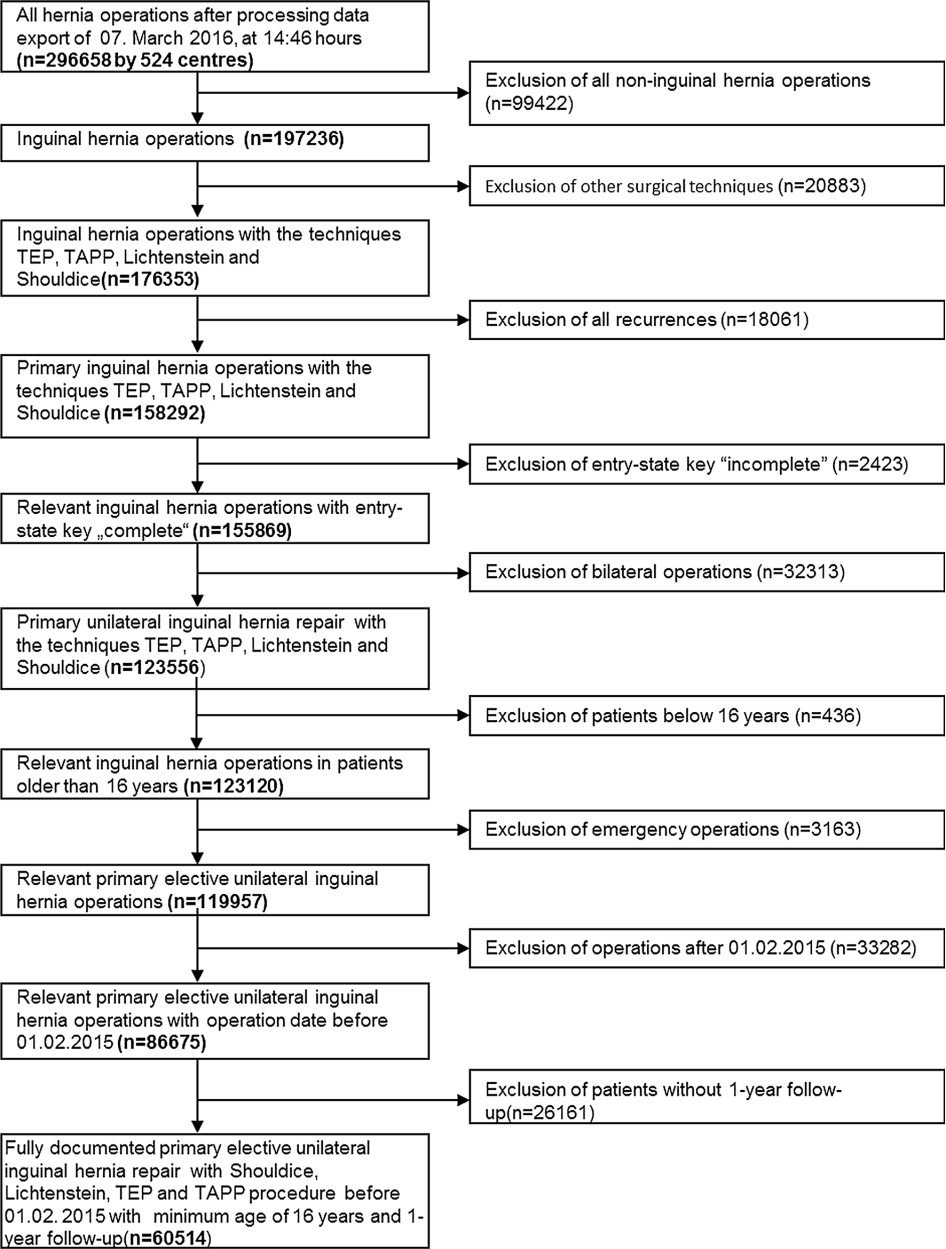
Flowchart of patient inclusion

The main inclusion criteria were a minimum age of 16 years, primary unilateral inguinal hernia, Shouldice, Lichtenstein, TEP or TAPP technique and availability of data at one-year follow-up (Fig. 1). In total, 60,514 patients were selected between September 1, 2009, and February 1, 2015. Of these patients, 2608 had been operated on with the Shouldice, 22,111 with the Lichtenstein, 14,559 with TEP and 21,236 with TAPP technique. Pairwise propensity score matching analyses were performed for these 60,514 patients to obtain homogeneous comparison groups, each. For the purpose of the present analyses the mutually independent matching groups Shouldice versus Lichtenstein, Shouldice versus TEP and Shouldice versus TAPP were thus formed.

### Statistical analysis

All analyses were performed with the software SAS 9.4 (SAS Institute Inc., Cary, NC, USA) and intentionally calculated to a full significance level of 5%, i.e., they were not corrected with respect to multiple tests, and each *p* value ≤0.05 represents a significant result. The perioperative and one-year follow-up outcome (intra- and postoperative complications, complication-related reoperations, pain at rest and on exertion, pain requiring treatment and recurrences at one-year follow-up) was compared for Shouldice versus Lichtenstein, TEP and TAPP techniques using, first of all, propensity score matching, each. Matched samples were then analyzed via McNemar’s test. As results the non-diagonal elements of the 2 × 2 frequency table, the corresponding p values and the odds ratio estimates with 95% confidence interval for matched samples are given. Propensity score (1:1) matching without replacement was performed using greedy algorithm and a caliper of 0.5 standard deviations. The variables used for matching were: age (years), gender (male/female), BMI (kg/m^2^), American Society of Anesthesiologists ASA Score (I–IV), preoperative pain (yes/no/unknown), defect size (European Hernia Society [EHS] classification Grade I < 1.5 cm, Grade II = 1.5–3 cm, Grade III > 3 cm) [16], defect localization (EHS classification medial, lateral, femoral, scrotal) [16], anticoagulant therapy with coumarin derivatives (yes/no) and antiplatelet therapy with platelet aggregation inhibitors (yes/no). The balance of the matched sample was checked using standardized differences (also given for the pre-matched sample) that should not exceed 10% (<0.1) after matching. For pairwise comparison of matching parameters between operation methods (for presenting the differences between the original—pre-matched—samples) Chi-square tests and *t* tests (Satterthwaite) were performed for categorical and continuous variables, respectively.

As sensitivity analyses, we estimated one multivariable logistic regression model per outcome variable based on all data available (*n* = 60,514) including the variables which had been chosen for matching.

## Results

### Shouldice versus Lichtenstein

Analysis of the variables used for matching when comparing the Shouldice versus Lichtenstein operations revealed significant differences prior to matching. For example, compared with their Lichtenstein counterparts, patients in the Shouldice group had a significantly lower age (41.2 ± 19.7 years vs. 64.6 ± 15.1 years; *p* < 0.001) and BMI (24.2 ± 3.5 vs. 25.8 ± 3.6; *p* < 0.001). Furthermore, in the Shouldice group the proportion of women was significantly larger (32.9 vs. 10.1%; *p* < 0.001), the hernia defects significantly smaller (*p* < 0.001; e.g., EHS I ≤ 1.5 cm 43 vs. 12.8%), the proportion of lateral and femoral inguinal hernias significantly larger, the proportion of scrotal inguinal hernias significantly smaller, the proportion of patients with preoperative pain significantly higher, the proportion of patients with continuing treatment with coumarin derivatives and with platelet aggregation inhibitors significantly lower and the proportion of patients with higher ASA score significantly lower.

**Fig. 2:**
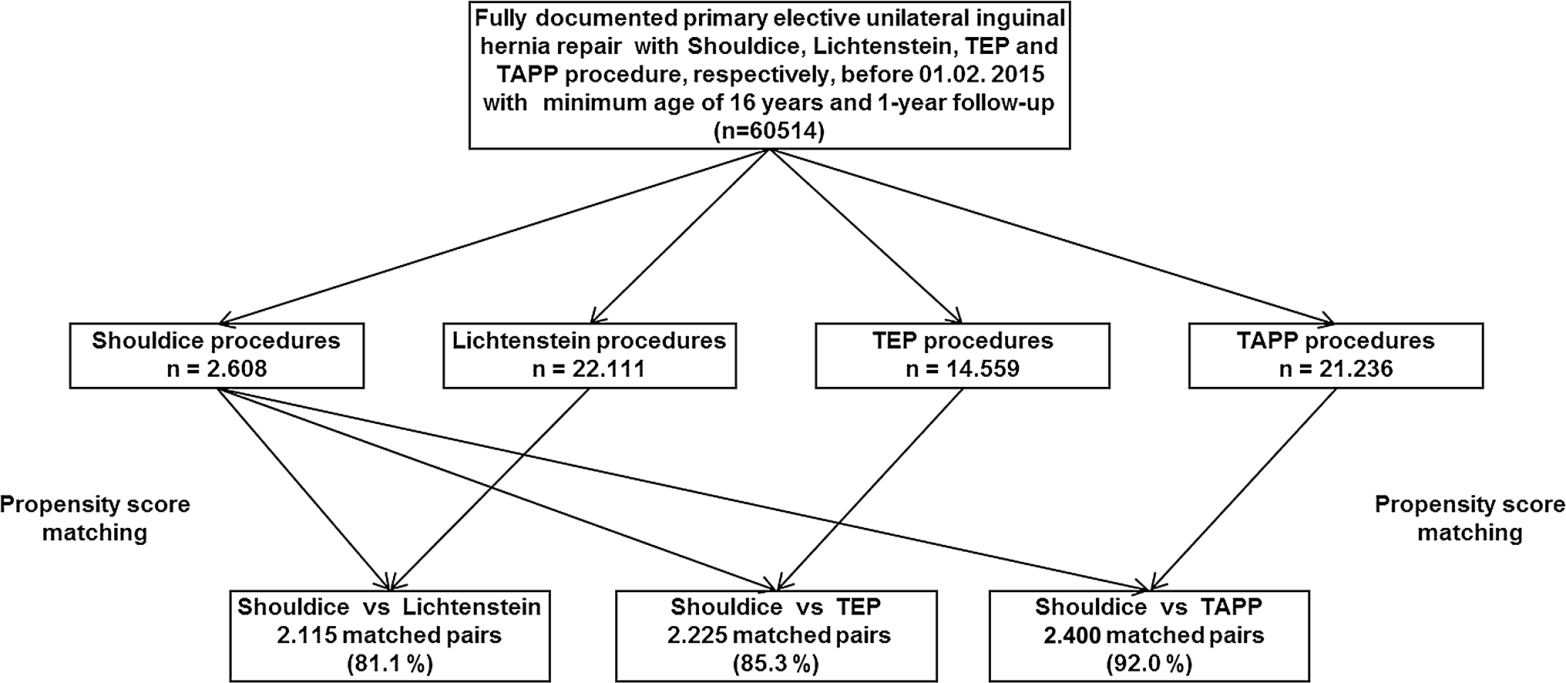
Flowchart of patient matching

Propensity score matching was applied to match the 2608 patients who had undergone a Shouldice operation with the 22,111 patients operated on with the Lichtenstein technique. Matching with the Lichtenstein population was successfully applied for 2115 (81.1%) of the Shouldice patients (Fig. 2).

**Fig. 3:**
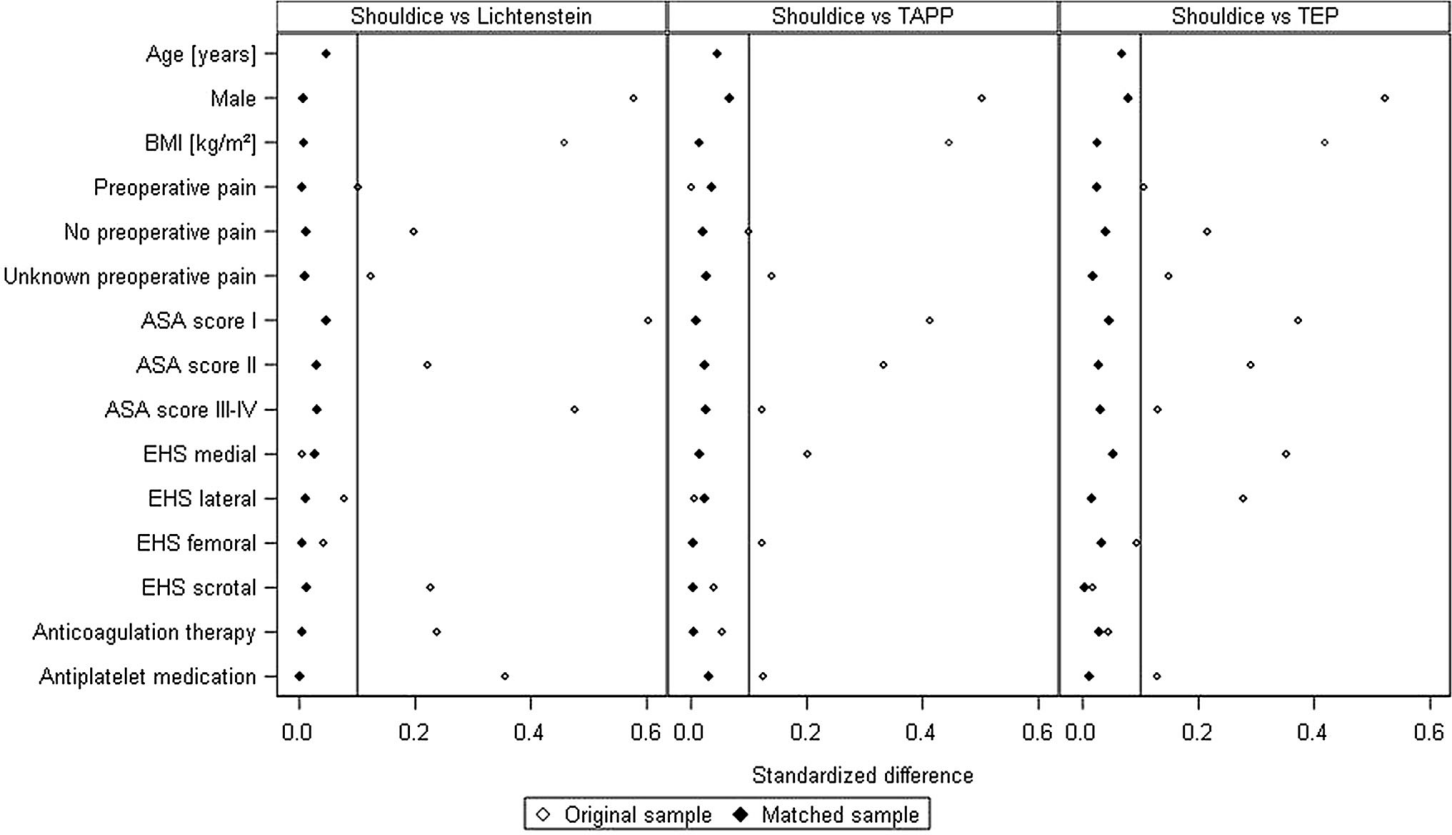
Standardized differences between the matching variables both before (original sample) and after matching (matched sample). *Standardized differences for age (original sample) are 1.333, 0.875 and 0.855 for Shouldice versus Lichtenstein, Shouldice versus TAPP and Shouldice versus TAP, respectively

Figure 3 illustrates the standardized differences between the matching variables both before (original sample) and after (matched sample) matching. After matching, the Shouldice and Lichtenstein collectives had a comparable age (44.8 ± 19.8 years vs. 45.7 ± 18.1 years), BMI (24.7 ± 3.4 vs. 24.7 ± 3.3), proportion of women (26 vs. 26.3%) and defect size (EHS I ≤ 1.5 cm 49.3 vs. 49.2%).

**Fig. 4:**
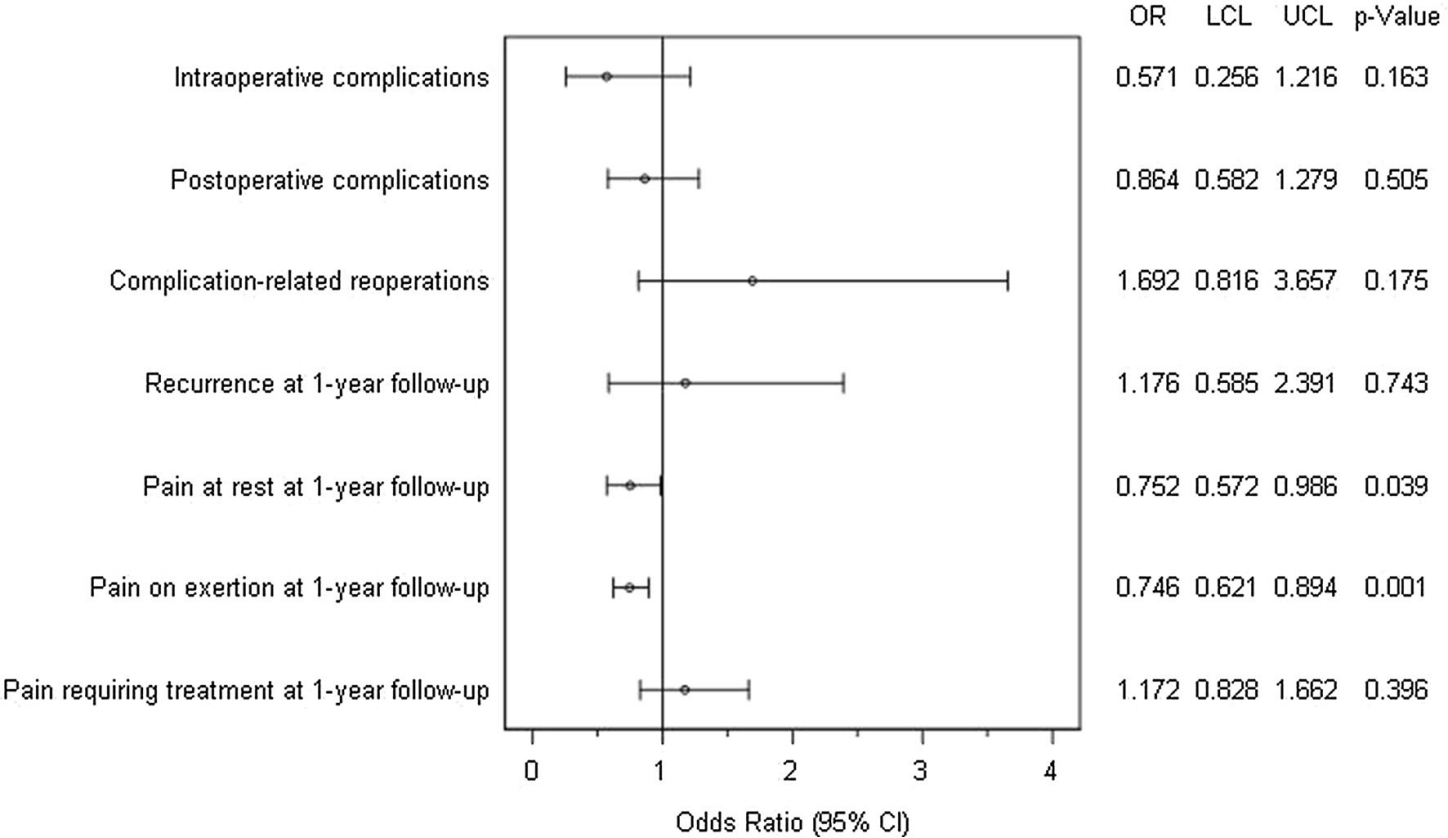
Results of matched-pair analyses of Shouldice versus Lichtenstein technique of the various outcome parameters

Figure 4 gives a summary of the results of matched-pair analyses for the two surgical techniques, Shouldice and Lichtenstein, for the various outcome parameters. Significant differences were found only for pain at rest and on exertion. A systematic deviation with regard to pain on exertion in favor of the Shouldice operation (10.0 vs. 13.4%; *p* = 0.001) was identified at one-year follow-up. That also applied for pain at rest at follow-up (4.6 vs. 6.1%; *p* = 0.039). No systematic deviation was detected for any of the other outcome variables between the Shouldice and Lichtenstein techniques.

### Shouldice versus TEP

Likewise, analysis of the variables used for matching when comparing Shouldice versus TEP revealed significant differences. Here, too, the patients in the Shouldice group had a significantly lower age (41.2 ± 19.7 vs. 56.4 ± 15.6 years; *p* < 0.001) and significantly lower BMI (24.2 ± 3.5 vs. 25.7 ± 3.5; *p* < 0.001). Furthermore, comparison of Shouldice versus TEP showed that in the Shouldice patient group the proportion of women was significantly higher (32.9 vs. 11.8%; *p* < 0.001), the hernia defects significantly smaller (*p* < 0.001; e.g., EHS I ≤ 1.5 cm 43.0 vs. 19.0%), the proportion of medial EHS classifications significantly larger, but that of lateral and femoral significantly smaller, the proportion of patients with preoperative pain significantly larger, the proportion of patients with continuing therapy with coumarin derivatives and with platelet aggregation inhibitors significantly lower and the proportion of higher ASA scores significantly lower.

Propensity score matching was applied to match the 2608 patients who had undergone a Shouldice operation with the 14,559 patients operated on with a TEP technique. Matching with the TEP population was successfully applied for 2225 (85.3%) of Shouldice patients (Fig. 2).

Figure 3 shows the standardized differences between the matching variables both before (original sample) and after (matched sample) matching. After matching, the Shouldice and TEP groups had a comparable age (43.7 ± 20.0 vs. 44.9 ± 16.9 years), BMI (24.5 ± 3.4 vs. 24.4 ± 3.4), proportion of women (27.3 vs. 30.8%) and defect size (EHS I ≤ 1.5 cm 48.6 vs. 49.5%).

**Fig. 5:**
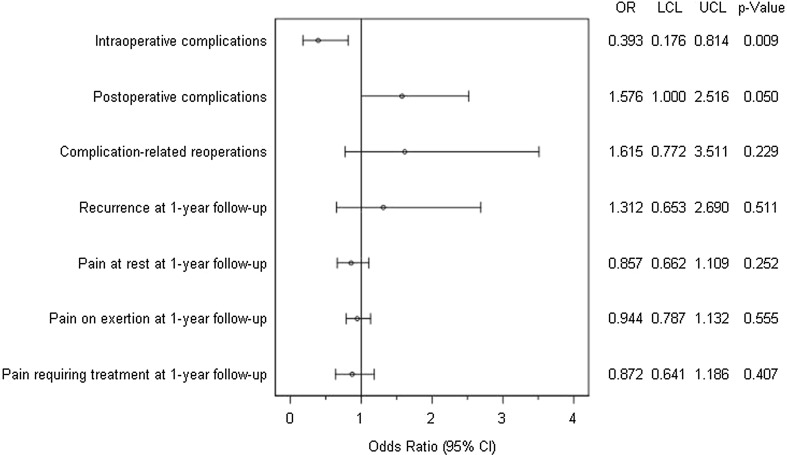
Results of matched-pair analyses of Shouldice versus TEP technique of the various outcome parameters

Figure 5 gives a summary of the results of matched-pair analyses for the two surgical techniques, Shouldice and TEP, for the various outcome parameters. A systematic deviation was noted between the two surgical techniques for the intraoperative and postoperative complications. For the intraoperative complications, a significant deviation was identified in favor of the Shouldice technique (0.5 vs. 1.3%; *p* = 0.009). Conversely, for the postoperative complications a slight deviation was detected in favor of TEP (1.5 vs. 2.3%; *p* = 0.050). No systematic discrepancy was detected between the two operative techniques for any of the other outcome variables.

### Shouldice versus TAPP

Analysis of the variables used for matching when comparing Shouldice versus TAPP also revealed significant differences. Significant differences were found, too, on comparing age (41.2 ± 19.7 vs. 56.7 ± 15.5 years; *p* < 0.001) and BMI (24.2 ± 3.5 vs. 25.8 ± 3.6; *p* < 0.001), with lower values identified for the Shouldice group. Furthermore, the Shouldice group compared with the TAPP group had a significantly larger proportion of women (32.9 vs. 12.5%; *p* < 0.001), significantly smaller hernia defects (*p* < 0.001; e.g., EHS I ≤ 1.5 cm 43.0 vs. 17.2%), a significantly larger proportion of medial and a significantly smaller proportion of femoral and scrotal hernias by EHS classification, a significantly smaller proportion of patients with no preoperative pain, a significantly smaller proportion of patients with continuing therapy with coumarin derivatives and with platelet aggregation inhibitors and a significantly smaller proportion of patients with higher ASA score.

Propensity score matching was applied to match the 2608 patients who had undergone a Shouldice operation with the 21,236 patients with a TAPP operation. Matching with the TAPP population was successfully applied for 2400 (92.0%) of the Shouldice patients (Fig. 2).

Figure 3 shows the standardized differences between the matching variables both before (original sample) and after (matched sample) matching. After matching, the Shouldice and TAPP collectives had a comparable age (42.6 ± 19.8 vs. 43.4 ± 16.6 years), BMI (24.4 ± 3.4 vs. 24.4 ± 3.7), proportion of women (28.8 vs. 31.8%) and defect size (EHS I ≤ 1.5 cm 46.6 vs. 45.1%).

**Fig. 6:**
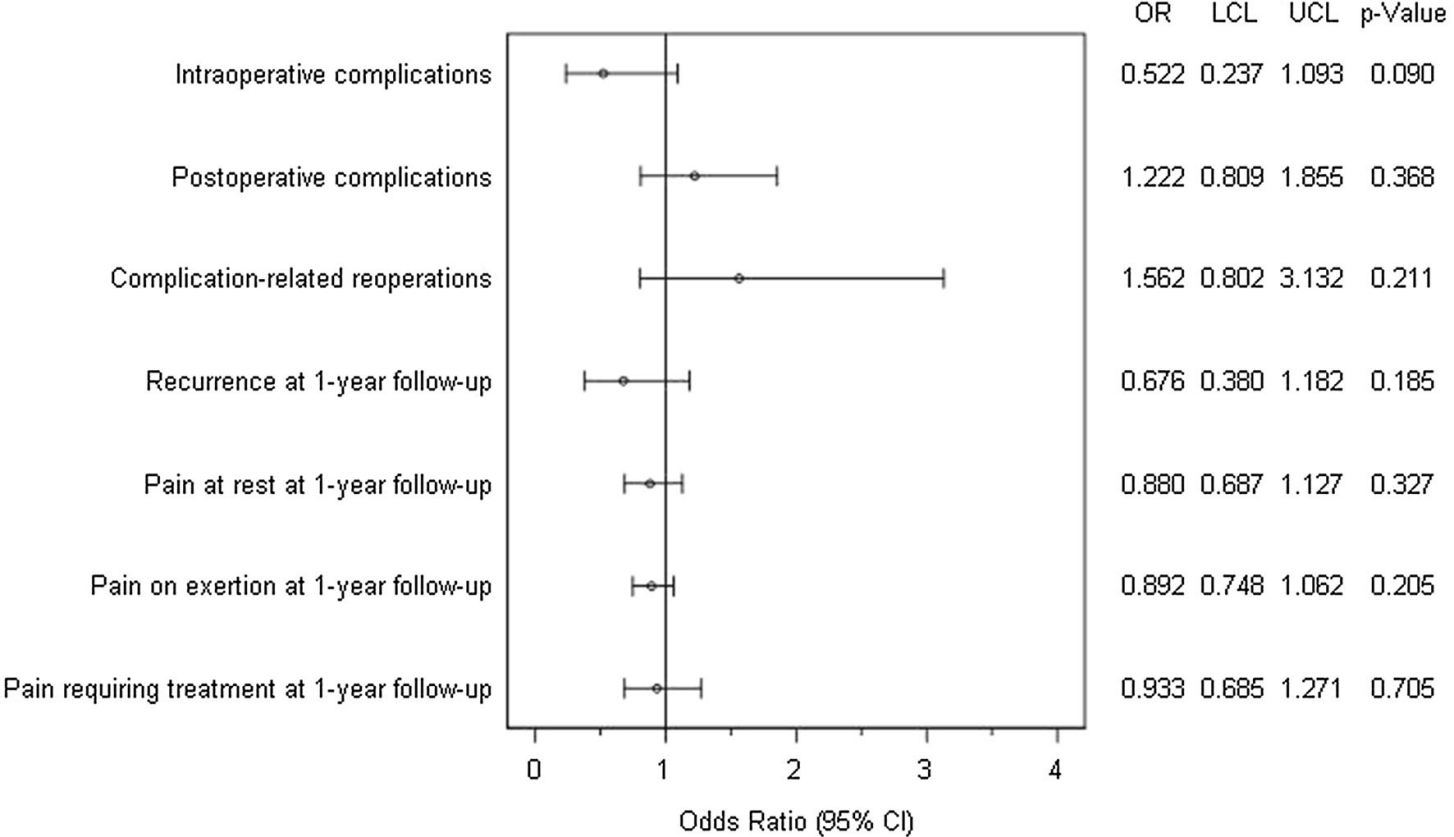
Results of matched-pair analyses of Shouldice versus TAPP technique of the various outcome parameters

Figure 6 gives a summary of the results of matched-pair analyses for the two surgical techniques, Shouldice and TAPP, for the various outcome parameters. No systematic deviation was identified between Shouldice and TAPP for any of the outcome variables.

### Sensitivity analyses

**Fig. 7:**
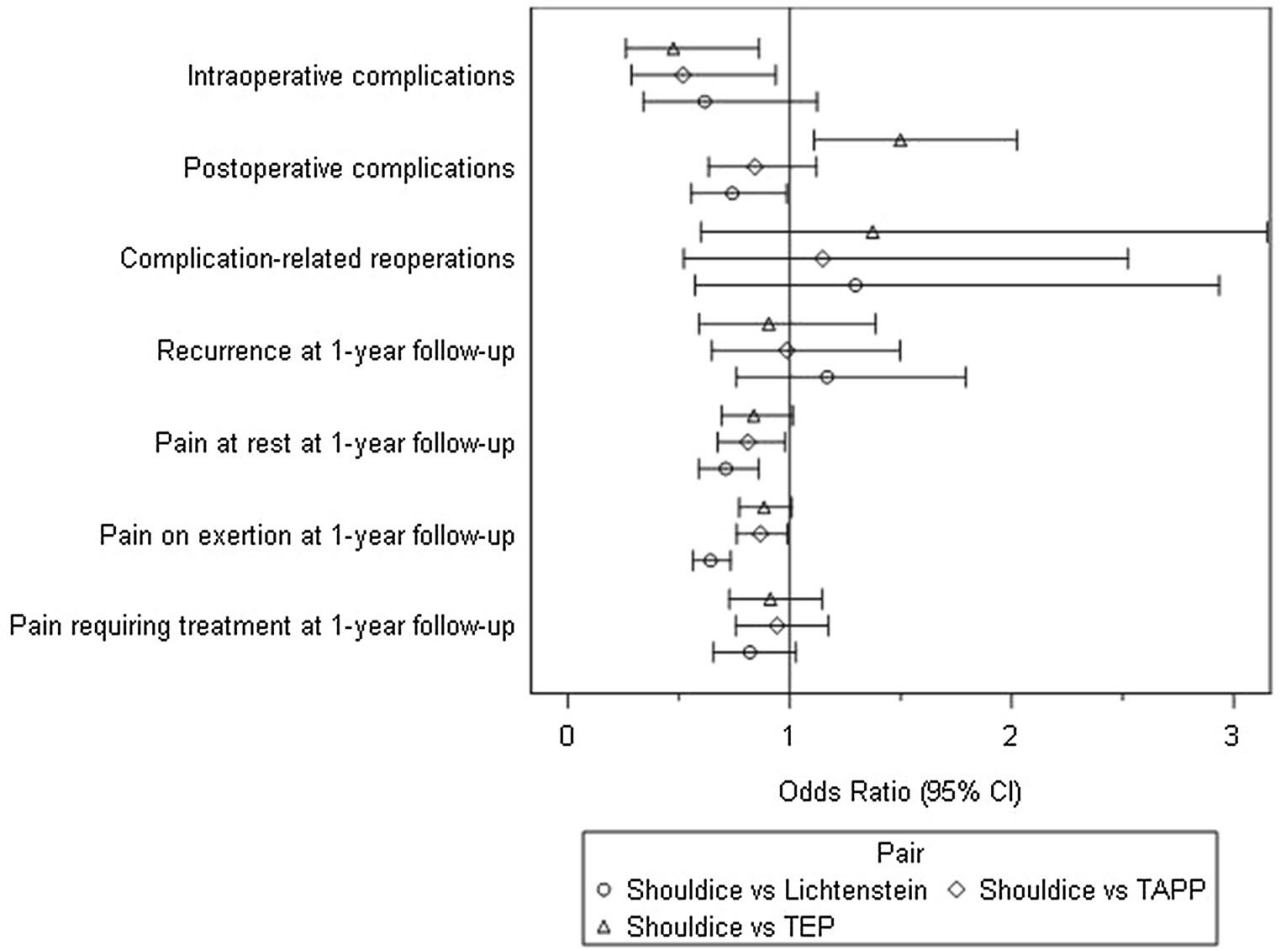
Results of multivariable models (pairwise OR estimates (95% CI))

Results are comprised in Fig. 7.

Pairwise estimates (OR estimate and its corresponding 95% confidence interval) for operation methods (Shouldice vs. {/Lichtenstein/TEP/TAPP}) are given. Model fit is a significant except for complication-related reoperations.

Sensitivity analyses verified our results; OR estimates are very close. But here—probably due to larger samples and a higher power—more significant effects in favor of the Shouldice method are found. This corresponds to the literature, and there can be no assurance that those significances are due to overestimation [17, 18].

## Discussion

The aim of the present propensity score analysis of data from the Herniamed Registry was to compare the surgical techniques of Shouldice versus Lichtenstein, Shouldice versus TEP and Shouldice versus TAPP with regard to intra- and postoperative complications, complication-related reoperations as well as recurrence and pain rates at one-year follow-up. To enhance comparability of these operative techniques, homogeneous comparison groups were first created for the different variables using propensity score matching. The variables used for matching were age, BMI, gender, ASA score, size of the hernia defect, EHS classification, preoperative pain and continuing treatment with coumarin derivatives or platelet aggregation inhibitors.

Comparison of the Shouldice versus Lichtenstein operation revealed a relevant systematic deviance in favor of the Shouldice technique with significantly less pain at rest and on exertion at one-year follow-up.

Likewise, on comparing the Shouldice versus TEP a relevant systematic difference was detected in the intraoperative complications in favor of the Shouldice operation. However, postoperative complications were more common on using the Shouldice technique.

On comparing the Shouldice with the TAPP technique, no systematic difference was noted for any of the outcome parameters.

In a multivariable logistic regression model as sensitivity analyses even more significant effects in favor of the Shouldice technique are found. But this needs to be carefully interpreted, because it can be an effect of larger samples, a higher power or even overestimation [17, 18].

The results presented here are based on the described patient collective which was formed as per the typical characteristics of the matching variables. The Shouldice patient collective was characterized by younger patients with a mean age of 40 years, larger proportion of women of around 30%, mean BMI value of 24 and a proportion of EHS I (<1.5 cm) and EHS II (1.5–3 cm) defect sizes of more than 85%. Besides, risk factors such as high ASA score and continuing treatment with coumarin derivatives and with platelet aggregation inhibitors were significantly less common in the Shouldice group. As such, the patients operated on with the Shouldice technique, as documented by the Herniamed Registry, tended to be younger, slimmer, with smaller defects and no risk factors. The Herniamed data now demonstrate that this selected patient group can be operated on with a good outcome with the Shouldice technique and with no evidence of any major disadvantages coming to light up to the end of first postoperative year compared with TAPP. The Shouldice technique was even found to have advantages over the Lichtenstein operation thanks to lower rates of pain at rest and on exertion at one-year follow-up. Compared with TEP, the intraoperative complication rate was significantly lower, but the postoperative complication rate was somewhat higher. Similarly, an Austrian prospective randomized control trial did not find any significant difference between the Shouldice, Bassini, Lichtenstein, TEP and TAPP surgical techniques with regard to the recurrence rate and complications up to three years following surgery [19].

A survey of patients from the Danish and Swedish Hernia Registry which compared 630 Shouldice and 1250 Lichtenstein patients with indirect inguinal hernia in young males identified a significantly lower pain rate for the Shouldice patients and no difference in the rate of new onset of inguinal protrusions [20]. Likewise, a Spanish prospective randomized trial identified comparable outcomes for the Shouldice and Lichtenstein techniques [21]. A prospective randomized trial that compared Shouldice versus TEP for primary unilateral inguinal hernia in men did not find any significant differences, apart from a longer operative time in the TEP group, between the two methods with regard to perioperative complications, hospital stay, recurrences or pain in the groin [22].

The largest prospective randomized trial with 1042 patients, carried out in Sweden, which compared the Shouldice versus TAPP techniques, did not find any differences in the complication rates [23]. Nor was any significant difference identified in the recurrence rates after 5 years [24]. There was also no difference between late discomfort at a 5-year follow-up after laparoscopic TAPP and Shouldice repair [25].

Therefore, while certain prospective randomized studies corroborate the comparative findings identified in this analysis of registry data for the Shouldice, Lichtenstein, TEP and TAPP surgical techniques for repair of primary unilateral inguinal hernias, some of the meta-analyses noted different results [26, 27]. The meta-analysis comprising 27 prospective randomized trials found a significantly higher total morbidity and chronic groin pain rate for the Shouldice technique compared with the laparo-endoscopic operation [26]. There were no differences regarding the incidence of hernia recurrence [26]. A Cochrane review [27] comparing 2566 Shouldice repairs with 1121 open mesh and 1608 other non-mesh techniques showed a higher recurrence rate for the Shouldice technique compared with other mesh techniques (OR 3.80, 95% CI 1.99–7.26), but a lower recurrence rate compared with other non-mesh techniques (OR 0.62, 95% CI 0.45–0.85). There were no significant differences in chronic pain and complications [27]. The corresponding guidelines were then formulated based on these meta-analyses [1–6]. However, the stance taken in the guidelines in favor of mesh-based techniques in inguinal hernia surgery is the focus of controversial debate in the literature [9], since an inguinodynia rate of over 21% has been reported for mesh procedures.

Incorrect or missing data limit a registry. In the Herniamed Hernia Registry the following measurements are used to optimize data entry: signed contract with the responsible surgeon for data correctness and completeness, indication of missing data by the software, once again review of the perioperative outcome on one-year follow-up and control of the data entry by experts as part of the certification process of hernia centers. The best safeguard is to match the data against another registry, administrative data and/or the literature [28]. Due to the high selection of patients in the Shouldice group the findings presented here are only partially in concordance with the existing literature. Additionally, the follow-up is with one year relatively short in view of the time interval needed to find the real recurrence rate [29].

In summary, the data presented here from the Herniamed Registry demonstrate that under routine conditions and on selecting patients on the basis of the influence variables age, weight, gender, defect size, defect localization, preoperative pain and certain risk factors, outcomes comparable with those of the Lichtenstein, TEP and TAPP techniques can be achieved with the Shouldice operation for primary unilateral inguinal hernia repair. A ‘tailored approach’ can be used and should take into account the impact exerted by the variables of interest on the outcome. Hence, based on the results presented here, younger, non-overweight patients with defect sizes up to 3 cm and no other risk factors can be operated on with the Shouldice technique. Additional large prospective randomized trials are urgently needed for comparison of the Shouldice technique with the Lichtenstein, TEP and TAPP mesh-based operations recommended in the guidelines. Such studies must definitely take into account the variables that impact the outcome (age, BMI, gender, EHS defect size, EHS defect localization, ASA score, preoperative pain and continuing treatment with coumarin derivatives and with platelet aggregation inhibitors). Only in such way can comparative patient collectives be achieved for effective comparison of the methods used for primary unilateral inguinal hernia repair.

## References

[1] Simons MP, Aufenacker T, Bay-Nielsen M, Bouillot JL, Campanelli G, Conze J, de Lange D, Fortelny R, Heikkinen T, Kingsnorth A, Kukleta J, Morales-Conde S, Nordin P, Schumpelick V, Smedberg S, Smietanski M, Weber G, Miserez M (2009). European Hernia Society guidelines on the treatment of inguinal Hernia in adult patients. Hernia.

[2] Miserez M, Peeters E, Aufenacker T, Bouillot JL, Campanelli G, Conze J, Fortelny R, Heikkinen T, Jorgensen LN, Kukleta J, Morales-Conde S, Nordin P, Schumpelick V, Smedberg S, Smitanski M, Weber G, Simons P (2014). Update with level 1 studies of the European Hernia Society guidelines on the treatment of inguinal hernia in adult patients. Hernia.

[3] Bittner R, Arregui ME, Bisgaard T, Dudai M, Ferzli GS, Fitzgibbons RJ, Fortelny RH, Klinge U, Kockerling F, Kuhry E, Kukleta J, Lomanto D, Misra MC, Montgomery A, Morales-Conde Reinpold W, Rosenberg J, Sauerland S, Schug-Pass C, Singh K, Timoney MM, Weyhe D, Chowbey P (2011). Guidelines for laparoscopic (TAPP) and endoscopic (TEP) treatment of inguinal hernia [International Endohernia Society (IEHS)]. Surg Endosc.

[4] Bittner R, Montgomery MA, Arregui E, Bansal V, Bingener J, Bisgaard T, Buhck H, Dudai M, Ferzli GS, Fitzgibbons RJ, Fortelny RH, Grimes KL, Klinge U, Kockerling F, Kumar S, Kukleta J, Lomanto D, Misra MC, Morales-Conde S, Reinpold W, Rosenberg J, Singh K, Timoney M, Weyhe D, Chowbey P (2015). Update of guidelines on laparoscopic (TAPP) and endoscopic (TEP) treatment of inguinal hernia (International Endohernia Society). Surg Endosc.

[5] Poelman MM, van den Heuvel B, Deelder JD, Abis GSA, Beudeker N, Bittner R, Campanelli G, van Dam D, Dwars BJ, Eker HH, Fingerhut A, Khatkov I, Kockerling F, Kukleta JF, Miserez M, Montgomery A, Munoz Brands RM, Morales-Conde S, Muysoms FE, Soltes M, Tromp W, Yavuz Y, Bonjer HJ (2013). EAES consensus development conference on endoscopic repair of groin hernias. Surg Endosc.

[6] Simons MP, Aufenacker TJ, Berrevoet F, Bingener J, Bisgaard T, Bittner R, Bonjer HJ, Bury K, Campanelli G, Chen DC, Chowbey PK, Conze J, Cuccurullo D, de Beaux AC, Eker HH, Fitzgibbons RJ, Fortelny RH, Gillion JF, van den Heuvel BJ, Hope WW, Jorgensen LN, Klinge U, Köckerling F, Kukleta JF, Konate I, Liem AL, Lomanto D, Loos MJA, Lopez-Cano M, Miserez M, Misra MC, Montgomery A, Morales-Conde S, Muysoms FE, Niebuhr H, Nordin P, Pawlak M, van Ramshorst GH, Reinpold WMJ, Sanders DL, Sani R, Schouten N, Smedberg S, Smietanski M, Simmermacher RKJ, Tran HM, Tumtavitikul S, van Veenendaal N, Weyhe D, Wijsmuller AR (2018) International guidelines for groin hernia management. Hernia. 10.1007/s10029-017-1668-x 10.1007/s10029-017-1668-x PMC5809582 29330835

[7] Shouldice EB (2003). The Shouldice repair of groin hernias. Surg Clin N Am.

[8] Shouldice EB (2010). Surgery illustrated—surgical atlas the shouldice natural tissue repair for inguinal hernia. BJU Int.

[9] Fischer JE (2013). Hernia repair: why do we continue to perform mesh repair in the face of the human toll of inguinodynia?. Am J Surg.

[10] Shouldice Hospital (2016) www.shouldice.com/the-shouldice-hernia-repair-surgery.html

[11] Malik A, Bell CM, Stukel TA, Urbach DR (2016). Recurrence of inguinal hernias repaired in a large hernia surgical specialty hospital and general hospitals in Ontario, Canada. Can J Surg.

[12] Stechemesser B, Jacob DA, Schug-Paß C, Köckerling F (2012). Herniamed: an internet-based registry for outcome research in hernia surgery. Hernia.

[13] Köckerling F, Simons T, Hukauf M, Hellinger A, Fortelny R, Reinpold W, Bittner R (2017). The importance of registries in the postmarketing surveillance of surgical meshes. Ann Surg.

[14] Lonjon G, Porcher R, Ergina P, Fouet M (2017). Boutron i potential pitfalls of reporting and bias in observational studies with propensity score analysis assessing a surgical procedure. Ann Surg.

[15] Haapaniemi S, Nilsson E (2002). Recurrence and pain three years after groin hernia repair. Validation of postal questionnaire and selective physical examination as a method of follow-up. Eur J Surg.

[16] Miserez M, Alexandre JH, Campanelli G, Corcione F, Currurullo D, Pascual MH, Hoeferlin A, Kingsnorth AN, Mandala V, Palot JP, Schumpelick V, Simmermacher RK, Stoppa R, Flament JB (2007). The European hernia society groin hernia classification: simple and easy to remember. Hernia.

[17] Martens EP, Pestman WR, de Boer A, Belitser SV, Klungel OH (2008). Systematic differences in treatment effect estimates between propensity score methods and logistic regression. Int J Epidemiol.

[18] Shah BR, Laupais A, Hux JE, Austin PC (2005). Propensity score methods gave similar results to traditional regression modeling in observational studies: a systematic review. J Clin Epidemiol.

[19] Pokorny H, Klingler A, Schmid T, Fortelny R, Hollinsky C, Kawji R, Steiner E, Pernthaler H, Függer R, Scheyer M (2008). Recurrence and complications after laparoscopic versus open inguinal hernia repair: results of a prospective randomized multicenter trial. Hernia.

[20] Bay-Nielsen M, Nilsson E, Nordin P, Kehlet H (2004). Chronic pain after open mesh and sutured repair of indirect inguinal hernia in young males. Br J Surg.

[21] Porrero JL, Bonachia O, López-Buenadicha A, Sanjuanbenito A, Sánchez-Cabezudo C (2005). Repair of primary inguinal hernia: Lichtenstein versus Shouldice techniques. Prospective randomized study of pain and hospital costs. Cir Esp.

[22] Wennström J, Berggren P, Akerud L, Järhult J (2004). Equal results with laparoscopic and Shouldice repairs of primary inguinal hernia in men. Report from a prospective randomized study. Scand J Surg.

[23] Berndsen F, Arvidsson D, Enander LK, Leijonmarck CE, Wingren U, Rudberg C, Smedberg S, Wickbom G, Montgomery A (2002). Postoperative convalescence after inguinal hernia surgery: prospective randomized multicenter study of laparoscopic versus shouldice inguinal hernia repair in 1042 patients. Hernia.

[24] Arvidsson D, Berndsen FH, Larsson LG, Leijonmarck CE, Rimbäck G, Rudberg C, Smedberg S, Spangen L, Montgomery A (2005). Randomized clinical trial comparing 5-year recurrence rate after laparoscopic versus Shouldice repair of primary inguinal hernia. Br J Surg.

[25] Berndsen FH, Petersson U, Arvidsson D, Leijonmarck CE, Rudberg C, Smedberg S, Montgomery A (2007). Discomfort five years after laparoscopic and Shouldice inguinal hernia repair: a randomized trial with 867 patients. A report from the SMIL study group. Hernia.

[26] Bittner R, Sauerland S, Schmedt CG (2005). Comparison of endoscopic techniques vs Shouldice and other open nonmesh techniques for inguinal hernia repair: a meta-analysis of randomized controlled trials. Surg Endosc.

[27] Amato B, Moja L, Panico S, Persico G, Rispoli C, Rocco N, Moschetti I (2012). Shouldice technique versus other open techniques for inguinal hernia repair. Cochrane Database Syst Rev.

[28] Hannan EL, Cozzens K, King SB (2012). The New York State Cardiac Registries: history, contributions, limitations, and lessons for future efforts to assess and publicly report healthcare outcomes. JACC.

[29] Köckerling F, Koch A, Lorenz R, Schug-Pass C, Stechemesser B, Reinpold W (2015). How long do we need to follow-up our hernia patients to find the real recurrence rate?. Front Surg.

